# COVID-19 and international primary care systems: Rebuilding a stronger primary care

**DOI:** 10.3399/bjgpopen20X101130

**Published:** 2020-09-09

**Authors:** Luke N Allen, Hajira Dambha-Miller

**Affiliations:** 1 Guest Editor and Editorial Board Member, BJGP Open, London, UK; 2 GP Academic Clinical Fellow, Nuffield Department of Primary Care Health Sciences, University of Oxford, Oxford, UK; 3 Editor-in-Chief, BJGP Open, London, UK

**Keywords:** International, Service organisation

In this BJGP Open collection, we examine how international primary care systems have performed in response to COVID-19. The past few months have seen rapid change and adaption of primary care systems to meet unprecedented new challenges. The pandemic has also exposed critical weaknesses in systems all over the world.^1^

The World Health Organization (WHO) and the World Organization of Family Doctors (WONCA) contend that an ideal primary care system should fulfil the core characteristics laid out by Barbara Starfield:^2^ primary care should be accessible, affordable, available at the time of need, and represent the ‘front-door’ to the health system. It should provide ongoing care and coordination with other specialists and focus on individuals and their families beyond their biological disease.^3,4^ The Astana Declaration^5^ and the WHO *Vision for Primary Health Care in the 21^st^ Century* ^6^ also make a case for empanelment (working for a listed or geographically defined population) and a model of primary care that is engaged with population health and the wider social determinants of health.^7,8^

Using a questionnaire that aligned with the Starfield principles, Goodyear-Smith and colleagues survey 1035 primary care leaders across 111 countries to assess the extent to which domestic primary care systems meet these standards.^9^ Few countries scored well in every domain, and those perceived as having a prepared pandemic plan and a strong primary care system still faced high rates of COVID-19 mortality. This important benchmarking study highlights the fact that all nations have room to improve.

Our second article in this collection is from Huston and colleagues, who provide a commentary on the primary care response to COVID-19 in six well-resourced countries: Australia, New Zealand, Canada, the Netherlands, the UK and the US.^10^ Their work identifies the specific areas where COVID-19 has directly spurred progress, as well as exposing latent weaknesses and even reversing gains made over previous decades. The pandemic has deepened integration between primary care and public health services, improved access by expediting the adoption of telemedicine, and forced providers to proactively engage with local epidemiological data. However, the authors also observe that the pandemic has disrupted the coordination of non-COVID services and the management of chronic conditions, constrained access for those unable to use phone or video-calling, and forced many fee-for-service centres to close or furlough staff in the US and Canada.

We then present an article from a low-income country, Cameroon. Ngo Bibaa shares her primary care experiences on the challenges of managing COVID-19 in rural Africa.^11^ Improvements to access through video-consulting and telemedicine have not been logistically possible. Basic personal protective equipment has been unobtainable. However, information dissemination and training systems have been working well, allowing the rapid adoption of infection control and prevention measures across the country. The author proposes a framework for what the 'new normal' should look like, using pandemic response to advance the development of strong primary care along the Starfield and broader Astana Declaration principles. In Ngo Bibaa’s model, all primary care functions are positioned to address wider social determinants of health with better integration into local communities and the broader health system. Although this model is proposed specifically for Cameroon, it aligns closely with the ‘three pillars’ of primary care; empowered people and communities; multisectoral policy and action; and integrated primary care and public health as the core of health services.^12^ This work also resonates with calls for European primary care to engage more deeply with social determinants in Europe,^13^ and the new conceptual framework on the levels of engagement with the social determinants in the upcoming WHO Bulletin special issue on primary health care.^14^

COVID-19 has revealed the areas where progress is needed most. Opportunities to improve coordination with public health and other specialities, as well as expanding access through telemedicine, extended opening hours, and new community clinics should not be misspent. The excess non COVID-19 deaths observed in many countries^15^ should prompt deeper investment in chronic management, improved prevention strategies, and more integrated, team-based primary care. The waiving or reduction of co-payments and fees that have been observed around the world (including in the Democratic Republic of Congo, Tanzania, Bangladesh, Sri Lanka, and Malaysia)^16^ should be capitalised upon to advance the Universal Health Coverage mandate of comprehensive services without financial hardship.^17^

COVID-19 has shone an uncomfortable spotlight on primary care over the past few months, exposing weaknesses even in the systems commonly held up as paragons of excellence. As primary care is so central to pandemic preparedness, response, and recovery,^18^ as well as attaining healthy and equitable societies^19,20^ it is absolutely imperative that we get it right; financing, resourcing, and structuring services that deliver on the Astana principles that were unanimously endorsed in 2018. Now is the time to rebuild primary care, stronger than ever.
